# Evaluation of SPP1/osteopontin expression as predictor of recurrence in tamoxifen treated breast cancer

**DOI:** 10.1038/s41598-020-58323-w

**Published:** 2020-01-29

**Authors:** Anna Göthlin Eremo, Kajsa Lagergren, Lana Othman, Scott Montgomery, Göran Andersson, Elisabet Tina

**Affiliations:** 1 Department of Clinical Research Laboratory, Faculty of Medicine and Health, Örebro University, Örebro, Sweden; 2 School of Medical Sciences, Faculty of Medicine and Health, Örebro university, Örebro, Sweden; 3 Clinical Epidemiology and Biostatistics, School of Medical Sciences, Örebro University, Örebro, Sweden; 4 Clinical Epidemiology Division, Karolinska Institutet, SE-171 76 Stockholm, Sweden; 5 Department of Epidemiology and Public Health, University College London, 1-19 Torrington Place, London, WC1E 7HB United Kingdom; 6 Division of Pathology, Department of Laboratory Medicine, Karolinska Institutet and Karolinska University Hospital Huddinge, S-141 86 Huddinge, Sweden

**Keywords:** Predictive markers, Molecular medicine, Breast cancer

## Abstract

Breast cancer patients treated with tamoxifen may experience recurrence due to endocrine resistance, which highlights the need for additional predictive and prognostic biomarkers. The glyco-phosphoprotein osteopontin (OPN), encoded by the SPP1 gene, has previously shown to be associated with poor prognosis in breast cancer. However, studies on the predictive value of OPN are inconclusive. In the present study, we evaluated tissue SPP1 mRNA and OPN protein expression as markers of recurrence in estrogen receptor- positive (ER+) breast cancer tissue. Tamoxifen- treated patients with recurrence or non-recurrence were selected using a matched case-control design. SPP1 mRNA expression was analysed using qPCR (n = 100) and OPN protein by immunohistochemistry (n = 116) using different antibodies. Odds ratios were estimated with conditional logistic regression. The SPP1 expression increased the risk of recurrence with an odds ratio (OR) of 2.50 (95% confidence interval [CI]; 1.30–4.82), after adjustment for tumour grade, HER 2 status and other treatments to OR 3.62 (95% CI; 1.45–9.07). However, OPN protein expression was not associated with risk of recurrence or with SPP1-gene expression, suggesting SPP1 mRNA a stronger prognostic marker candidate compared to tumor tissue OPN protein.

## Introduction

**Figure 1:**
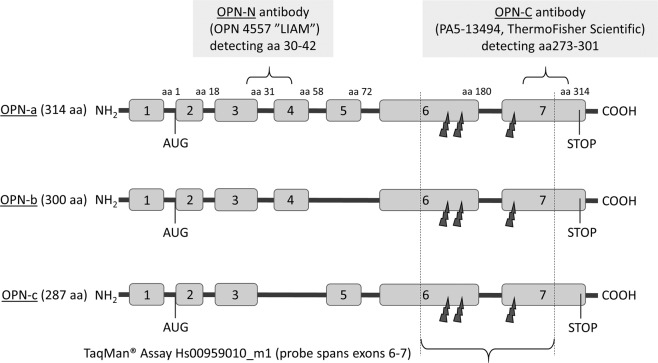
The protein isoforms OPN-a, OPN-b and OPN-c with corresponding exons. The short curly brackets show the sites of binding of the different antibodies for OPN-N and OPN-C, and the dark grey arrows show cleavage sites for MMPs and thrombin. The TaqMan-assay detects all transcripts as all transcript splice variants include exon 6 and 7 (long curly bracket).

Breast cancer is a heterogeneous disease and the majority of tumours express oestrogen receptors (ER). Patients with ER positive (ER+) breast cancer are candidates for endocrine treatment, such as tamoxifen, although up to 30% of women are expected to experience recurrence due to *de novo* or acquired tamoxifen resistance^1^. Osteopontin (OPN) is a multifunctional secreted integrin-binding glycoprotein, which has been suggested to have a prognostic value and to be involved in the tamoxifen response^2^. The protein is encoded by the secreted phosphoprotein 1 (SPP1) gene, located on chromosome 4 (4q13). As the SPP1 gene transcript is subject to alternative splicing and the OPN protein to post-translational modifications such as proteolytic processing, glycosylation, tyrosine sulfation and serine/threonine phosphorylation, OPN occurs in several variants. The intracellular variant (iOPN) lacks the N-terminal signal sequence and remains in the cytoplasm. The secreted OPN variants (sOPN) include OPN-a, the full-length protein with a total of 7 exons, and the two splice variants OPN-b and OPN-c, lacking exon 5 (aa 59–72) and 4 (aa 31–57) respectively^3^ (Fig. 1). Cleavage by proteases (e.g. thrombin, MMP3 and/or MMP7) produces OPN-N (N-terminal fragment) and OPN-C (C-terminal fragment)^4^. The OPN functions are linked to various physiological and pathological events. Several studies have shown a role of OPN in carcinogenesis, mostly by supporting migratory behaviour in tumour cells and regulating the tumour microenvironment in favour of metastasis^5–9^. In addition, OPN promotes epithelial-mesenchymal transition during metastasis, further indicating an important role in cancer progression^10,11^. The clinical utility is recognized in the CancerSeek test, screening for up to eight solid tumours using liquid biopsies, which include OPN as one of eight protein markers^12^.

Studies have shown that OPN protein overexpression in breast tumours, compared to normal breast tissue, and higher plasma levels of OPN are positively associated with increased tumour burden and shorter survival of patients^13,14^. In a meta-analysis of 10 clinical studies, comprising a total of 1,567 participants, both high level of serum and tissue OPN indicated a poor breast cancer outcome. In addition, the isoform OPN-c was more reliably associated with worse prognosis than the full-length OPN-a^15^. Exon 4 of OPN has been described to predict a favourable response to tamoxifen although the predictive value of OPN remains elusive with regard to post-translational modifications, splicing variants and subcellular localization^2^. Here, we further investigate the significance of SPP1 and OPN (C- and N-terminals) in predicting recurrence among tamoxifen- treated patients with ER+ breast cancer.

## Materials and Methods

### Patients

The study base included all women diagnosed with breast cancer at the Department of Oncology, Örebro University Hospital, Sweden, between January 1, 2000 and December 31, 2010 (n = 1 696). The patients were identified retrospectively from the Regional Cancer Centre (RCC) Uppsala - Örebro from where information was retrieved on primary tumour characteristics (biological markers, size, histology and axillary lymph node status) and clinical data (postoperative treatment and time of recurrence). For selection of the patients who had undergone primary surgery from which spare fresh frozen tumour tissue was stored, the list of patients obtained from RCC was cross-checked against the biobank database at the Department of Pathology, Örebro University Hospital (n = 712). With a focus on endocrine resistance, patients who had an ER+ tumour, were treated with adjuvant tamoxifen and had no metastasis at the time of diagnosis were identified (n = 316). Follow-up data from RCC by March 31, 2013, identified 36 patients with distant metastasis (=endpoint) while 280 patients remained recurrence-free. The patients having recurrence within 24 months from diagnosis were excluded from the study (n = 9). The remaining patients with distant recurrence (n = 27) were each assigned a risk set number and the following matching criteria were recorded; primary tumour size (all tumour sizes in mm were ranked smallest to largest and grouped 1–5 based on 20^th^ percentile distribution), menopausal status (pre- postmenopausal), time to recurrence (months) and lymph node dissemination (yes/no). Any recurrence-free patients matching the features of a risk set were eligible for random selection into a risk set. One to five matched controls were randomly selected into each risk set and a total of 130 breast cancer patients were included (recurrent n = 27, non-recurrent n = 103) After exclusion of patients with no written consents (n = 10) and poor tissue quality (n = 4), 27 patients with recurrence and 89 non-recurrent patients remained. The median follow-up time for all 116 patients were 96 months. The patients and tumour characteristics are described in Table 1.

**Table 1:** Characteristics for the original matched study patients (N = 130) and the patients with SPP1 gene expression data measured by qPCR (N = 100) and OPN protein expression (N = 116). SD = standard deviation, PR = Progresterone receptor, HER2 = Human epidermal growth factor receptor 2, Rad = Radiation therapy Cyt = Cytostatic drugs, n.a. = non available.

	Total	Orignial cohort (N = 130)		p-value^a^	SPP1 gene expression (N = 100)		p-value^a^	OPN protein expression (N = 116)		p-value^a^
		Recurrence	Non-recurrence		Recurrence	Non-recurrence		Recurrence	Non-recurrence	
		N (column %)	N (column %)		N (column %)	N (column %)		N (column %)	N (column %)	
		27 (100)	103 (100)		25 (100)	75 (100)		27 (100)	89 (100)	
Mean age at diagnosis (years ± SD)	56.6 ( ± 12.8)	63.5 ( ± 14.6)	0.027^c^	56.9 ( ± 13.2)	63.7 ( ± 14.3)	0.038^c^	56.6 ( ± 12.8)	65.3 ( ± 14.2)	0.005^c^	
Mean tumour size (mm ± SD)	36.4 ( ± 29.6)	28.3 ( ± 18.0)	0.18^d^	34.1 ( ± 29.6)	26.7 ( ± 16.4)	0.24^d^	36.4 ( ± 29.6)	27.5 ( ± 17.0)	0.15^d^	
Tumour size by 20th percentile*	1 (10–15 mm)	5 (18.5)	20 (19.4)	0.65	5 (20.0)	15 (20.0)	0.69	5 (18.5)	17 (19.1)	0.39
	2 (16–20 mm)	5 (18.5)	24 (23.3)		5 (20.0)	19 (25.3)		5 (18.5)	21 (23.6)	
	3 (21–28 mm)	5 (18.5)	20 (19.4)		5 (20.0)	14 (18.7)		5 (18.5)	19 (21.3)	
	4 (30–35 mm)	3 (11.1)	19 (18.4)		3 (12.0)	15 (20.0)		3 (11.1)	18 (20.2)	
	5 (40–150 mm)	9 (33.3)	20 (19.4)		7 (28.0)	12 (16.0)		9 (33.3)	14 (15.7)	
Grade (Elston)	I	2 (7.4)	15 (14.6)	0.64	2 (8.0)	11 (14.7)	0.72	2 (7.4)	11 (12.4)	0.73
	II	16 (59.3)	60 (58.3)		14 (56.0)	41 (54.7)		16 (59.3)	54 (60.7)	
	III	9 (33.3)	28 (27.2)		9 (36.0)	23 (30.7)		9 (33.3)	24 (27.0)	
Menopausal status*	Pre-	11 (40.7)	25 (24.3)	0.097^b^	10 (40.0)	17 (22.7)	0.12^b^	11 (40.7)	17 (19.1)	0.038^b^
	Post-	16 (59.3)	78 (75.7)		15 (60.0)	58 (77.3)		16 (59.3)	72 (80.9)	
PR-Status	Negative	3 (11.1)	13 (12.6)	1.00/0.29^e^	3 (12.0)	11 (14.7)	1.00/0.39^e^	3 (11.1)	12 (13.5)	1.00/0.38^e^
	Positive	23 (85.2)	90 (87.4)		21 (84.0)	64 (85.3)		23 (85.2)	77 (86.5)	
	n.a.	1 (3.7)	0 (0.0)		1 (4.0)	0 (0.0)		1 (3.7)	0 (0.0)	
Lymph node infiltration*	No	8 (29.6)	35 (34.0)	0.82^b^	8 (32.0)	28 (37.3)	0.81^b^	8 (29.6)	32 (36.0)	0.65^b^
	≥ 1	19 (70.4)	68 (66.0)		17 (68.0)	47 (62.7)		19 (70.4)	57 (64.0)	
HER2	Negative	21 (77.8)	66 (64.1)	1.00/0.24^e^	21 (84.0)	57 (76.0)	1.00/0.60^e^	21 (77.8)	59 (66.3)	1.00/0.37^e^
	Positive	3 (11.1)	10 (9.7)		3 (12.0)	9 (12.0)		3 (11.1)	9 (10.1)	
	n.a.	3 (11.1)	27 (26.2)		1 (4.0)	9 (12.0)		3 (11.1)	21 (23.6)	
Aromatase inhibitors	No	26 (96.3)	90 (87.4)	0.30	24 (96.0)	64 (85.3)	0.29	26 (96.3)	77 (86.5)	0.29
	Yes	1 (3.7)	13 (12.6)		1 (4.0)	11 (14.7)		1 (3.7)	12 (13.5)	
Trastuzumab treatment	No	25 (92.6)	102 (99.0)	0.11	23 (92.0)	74 (98.7)	0.15	25 (92.6)	88 (98.9)	0.14
	Yes	2 (7.4)	1 (1.0)		2 (8.0)	1 (1.3)		2 (7.4)	1 (1.1)	
Additional treatment	No other	1 (3.7)	16 (15.5)	0.075	1 (4.0)	12 (16.0)	0.11	1 (3.7)	16 (18.0)	0.032
	Rad	9 (33.3)	46 (44.7)		9 (36.0)	34 (45.3)		9 (33.3)	41 (46.1)	
	Rad and cyt	17 (63.0)	41 (39.8)		15 (60.0)	29 (38.7)		17 (63.0)	32 (36.0)	

*Matching criteria, ^a^Fisher’s Exact Test if not else is specified (cells with expected counts < 5). ^b^Chi2-test (all cells with expected count > 5). ^c^Student’s T-test, equal variances assumed, ^d^Student’s T-test, equal variances not assumed (Lavene’s test p < 0.05). ^e^Chi2-test including cases with n.a.

### Tissue collection

After primary surgery, a pathologist examined and dissected material from the patients´ breast tissue for pathological anatomical diagnosis (PAD). Tissue samples from tumour areas were routinely snap frozen and stored at −80 °C.

### Isolation of RNA and cDNA synthesis

RNA was isolated from fresh frozen tumour tissues using the RNeasy Plus Micro Kit (Qiagen, Solna, Sweden). The RNA concentration was measured and quality checked using NanoDrop Spectrophotometer ND-1000 (NanoDrop Technologies, Thermo Fisher Scientific, Wilmington, DE, USA). Complementary DNA (cDNA) was synthesized from 400 ng RNA using High Capacity cDNA Reverse Transcription Kit (Applied Biosystems, Foster City, CA) in a 20 µL reaction by incubations at 25 °C for 10 min, 37 °C for 120 min, 85 °C for 5 s and 4 °C in a S1000 Thermal Cycler (BioRad, Stockholm, Sweden). The cDNA samples were stored at −20 °C.

### Gene expression of SPP1 by quantitative real-time PCR (qPCR)

Briefly, gene expression was measured by mixing cDNA (40 ng/µL) with TaqMan Gene Expression Assay (Applied Biosystems, Foster City, CA, USA) for the target gene SPP1 (Hs00959010_m1) and for the reference genes ABL1 and TOP1 (Hs00243257_m1, 01104728_m1) and TaqMan Fast Advanced Master Mix (Applied Biosystems) in 20 µL reactions. The TaqMan assay for SPP1 covers transcripts for both secreted and intracellular isoforms of OPN (NM_001040058.1, NM_000582.2, NM_001040060.1, NM_001251829.1 and NM_001251830.1, Fig. 1). The qPCR mixtures were set in duplicates in wells of 96-well plates and run using 7900HT Fast Real-Time PCR system (Applied Biosystems) at 50 °C for 2 min, 95 °C for 20 s and then 95 °C for 1 s followed by 60 °C for 20 s, repeated for 40 cycles. The quantification cycle (Cq) was set automatically. The expression of SPP1 was normalised against the mean value of the two reference genes (∆Cq) and the 2^-∆Cq^ values used for further analysis.

### Protein expression of OPN by immunohistochemistry (IHC)

Formalin-fixed and paraffin embedded (FFPE) tumour tissues (n = 116) were deparaffinized in TissueClear (Sakura Finetek Sweden AB, Gothenburg, Sweden), and rehydrated in decreasing series of ethanol concentrations ending in de-ionized (DI) H_2_O. The primary antibody used for detection of OPN C-terminal (OPN-C) was a polyclonal IgG antibody produced in rabbit against aa 273–301 (PA5-13494, ThermoFisher Scientific). The primary antibody for detection of OPN N-terminal (OPN-N) was a custom made polyclonal and raised in rabbit against aa 46–58 (OPN 4557 “Liam”) recognizing aa 30–42 in mature OPN-a, OPN-b and iOPN^16,17^ (Fig. 1). For OPN-C antigen retrieval was performed in Diva Decloaker buffer (pH 6) at 110 °C for 10 min in Decloaking Chamber^TM^ (Biocare Medical/Histolab, Gothenburg, Sweden). For OPN-N no antigen retrieval was required due to high background signal. The staining procedure was performed using the automated intelliPATH FLX staining instrument (Biocare Medical) and the HRP-polymer detection system MACH 1 (Biocare Medical). The antibodies were diluted (OPN-C 1:100 and OPN-N 1:800) in Da Vinci Green diluent (Biocare Medical/Histolab) and incubated for 30 min at room temperature (RT). The staining was visualised with 3,3′-Diaminobenzidine (DAB) followed by counterstaining with Mayer’s Haematoxylin (HTX) for 5 min at RT, tissue dehydration in ethanol and xylene and mounting using Pertex mounting medium (Histolab). The antibodies and the staining procedures were evaluated using tissue micro arrays of healthy controls from gall bladder, appendix, liver, kidney, thyroid, spleen, pancreas, lung, colon, small intestine, uterus, skeletal muscle and placenta. The OPN expression patterns in the different tissues were as expected according to prior publications^17,18^.

### Immunohistochemical scoring

The tissue slides were scanned for digitalization with Pannoramic 250 Flash II (3DHISTECH: Budapest, Hungary) and evaluated using the software CaseViewer version 2.0 (3DHISTECH). The evaluators (KL and AGE) were blinded to all clinical data and patient outcome. For each slide and staining, six separate areas (three tumour and three stroma) corresponding to x40 vision fields were analysed. In the three tumour areas, the percentage of tumour cells within each staining intensity category (0 = negative, 1+ = weak, 2+ = moderate and 3+ = strong) was recorded. An H-score for each tumour area was calculated using the algorithm [1 * (% cells 1+) + 2 * (% cells 2+) + 3 * (% cells 3+)] and the mean H-score was used for further statistical analysis [22]. The stromal staining was assessed by estimating the dominating staining intensity (0 = negative, 1 = weak, 2 = moderate, 3 = strong) multiplied with the stromal area coverage (0% = 0, <10% = 1, 10–50% = 2 and >50% = 3). The mean stromal staining score from the three areas, ranging between 0–9, were subsequently re-categorized into 0 (score 0.0–1.0), 1 (score 1.1–3.0), 2 (score 3.1–6.0) and 3 (score 6.1–9.0) and used for statistical analysis.

### Statistical analysis

The Saphiro-Wilk test was used in order to control data normality. The 2^−ΔCt^ values representing SPP1 gene expression were log-transformed in order to obtain normally distributed data and calculate standard deviation scores (z-scores). For protein expression, the H-score values were ordered numerically and divided into five equal sized groups based on 20^th^ percentiles. Also, the protein expression was categorized as OPN-C^high^, OPN-C^low^, OPN-N^high^ and OPN-N^low^ by using the median H-score value for each staining as cut-offs. The difference in tumour gene expression between patients with and without recurrence was calculated using Mann-Whitney U-test.

The main analysis was performed as a matched case-control study. Odds ratios (OR) with 95% confidence intervals (95% CI) were assessed using conditional logistic regression, with adjustment for radiation therapy, cytostatic drugs, HER2-status and tumour grade (Elston). Additionally, Kaplan-Meier curves with log-rank survival tests between patients with different OPN/SPP1-expression. The endpoints were categorized as 0 = no recurrence or 1 = distant metastasis. The patients were censored at the date of data withdrawal from RCC and the patients that died to other or unknown causes were censored at the date of death according to the Swedish death registry. The diagnostic ability of OPN gene expression (z-scores) was evaluated using Reciever Operating Characteristic (ROC) curve analysis, with Area Under the Curve (AUC), in comparison to other known diagnostic and prognostic markers (Elston grade, tumour size, lymph nodes and HER2-status). The relationship between SPP1 gene expression (log 2^−ΔCt^) and primary tumor expression of OPN-C and OPN-N (H-score) was tested with Spearman rang correlation test and linear regression. The Kruskal Wallis test was used to examine the distribution of SPP1 and stromal OPN expression among the groups OPN-C^high^/OPN-N^high^, OPN-C^high^/OPN-N^low^, OPN-C^low^/OPN-N^high^ and OPN-C^low^/OPN-N^low^ and Chi2 was used to test if patients with or without recurrence distributed differently between the groups. Statistical significance was defined as p-values < 0.05. Statistical analyses were performed using IBM SPSS Statistics Version 22, STATA version 14.2 and GraphPad Prism version 7.03.

### Approval

The local Ethics Committee in Uppsala, Sweden (Uppsala/Örebro No. 2011/070) approved the study.

### Accordance

All procedures performed in studies involving human participants were in accordance with the ethical standards of the institutional and/or national research committee and with the 1964 Helsinki declaration and its later amendments or comparable ethical standards.

### Informed consent

Informed consent was obtained from all individual participants still alive and included in the study. In accordance to the ethical approval, consents were not required from deceased patients’ relatives.

## Results

From the cohort of matched patients’ tumour tissues (n = 130), 100 were available for SPP1 gene expression analysis (by qPCR) and 116 were available for OPN protein analysis (by IHC). Age was not included in the matching procedure and the patients with recurrence, as a group, were younger at the time of diagnosis than non-recurrent patients (Table 1). However, the patients were matched on menopausal status into risk sets as a way of restricting biological variation caused by age. The age distribution of patients within each risk set are shown in Supplementary Figure 1. The pre-menopausal patients had a mean age of 45.1 (SD = 5.1) years and the post-menopausal patients a mean age of 68.6 (SD = 11.3) years (Student’s t-test, *p* < 0.0001). Nonetheless, the menopausal status differed between the 116 recurrent and non-recurrent patients having tumours analysed for OPN protein expression (Table 1). By using the matched case-control study design and conditional logistic regression analysis, the patients were compared within risk sets. Thus, the measure of risk was not influenced by group differences in menopausal status. Among these 116 patients, patients with recurrence received more treatment than non-recurrent when compared at group level (Table 1, *p* = 0.032). We adjusted for treatment in the statistical analysis with conditional logistic regression.

### SPP1 gene expression

**Figure 2:**
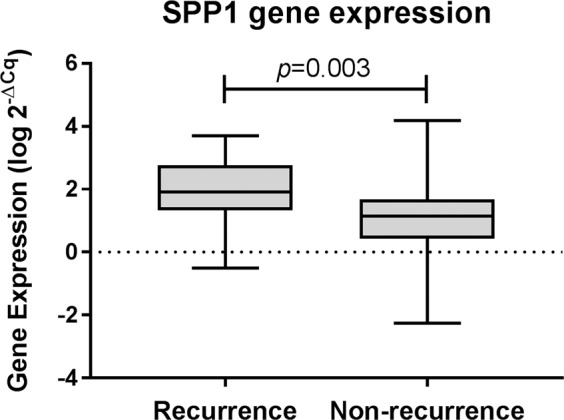
Difference in gene expression between tumours from breast cancer patients with and without distant metastasis (recurrence). Mann-Whitney U test p = 0.003.

RNA samples with concentrations< 40 ng/ µL (n = 15) were excluded from the study. Gene expression of SPP1 was analysed in 101 ER+ breast tumours and Ct-values were finally obtained from 100 tumours. The group of tumours from patients with recurrence (n = 25) expressed higher levels of SPP1 (*p* = 0.003, Fig. 2) than non-recurrence tumours (n = 75). The results from conditional logistic regression analysis indicated the risk of recurrence increased with OR = 2.50 (95% CI 1.30–4.82, *p* = 0.006) per standard deviation from the mean SPP1 gene expression. The OR for risk increased with multi regression analysis, adjusting for tumour grade, HER2-status and other treatments (3.62, 1.45–9.07; *p* = 0.006). The results from the additional log-rank survival tests confirm a statistically significant association of high SPP1-gene expression to shorter recurrence-free survival, shown in Kaplan-Meier curves in Supplementary Figure 2. As the SPP1 gene expression z-scores associated to recurrence, we investigated the diagnostic value of SPP1 using ROC-curves with AUC. The AUC for SPP1 was 0.70 (95% CI 0.58–0.83, *p* = 0.003). The AUC for the other tested characteristics were below 0.53 (presented in Table 1, Supplementary Information), which is anticipated due to the matched case-control design.

### Osteopontin (OPN) protein expression

**Figure 3:**
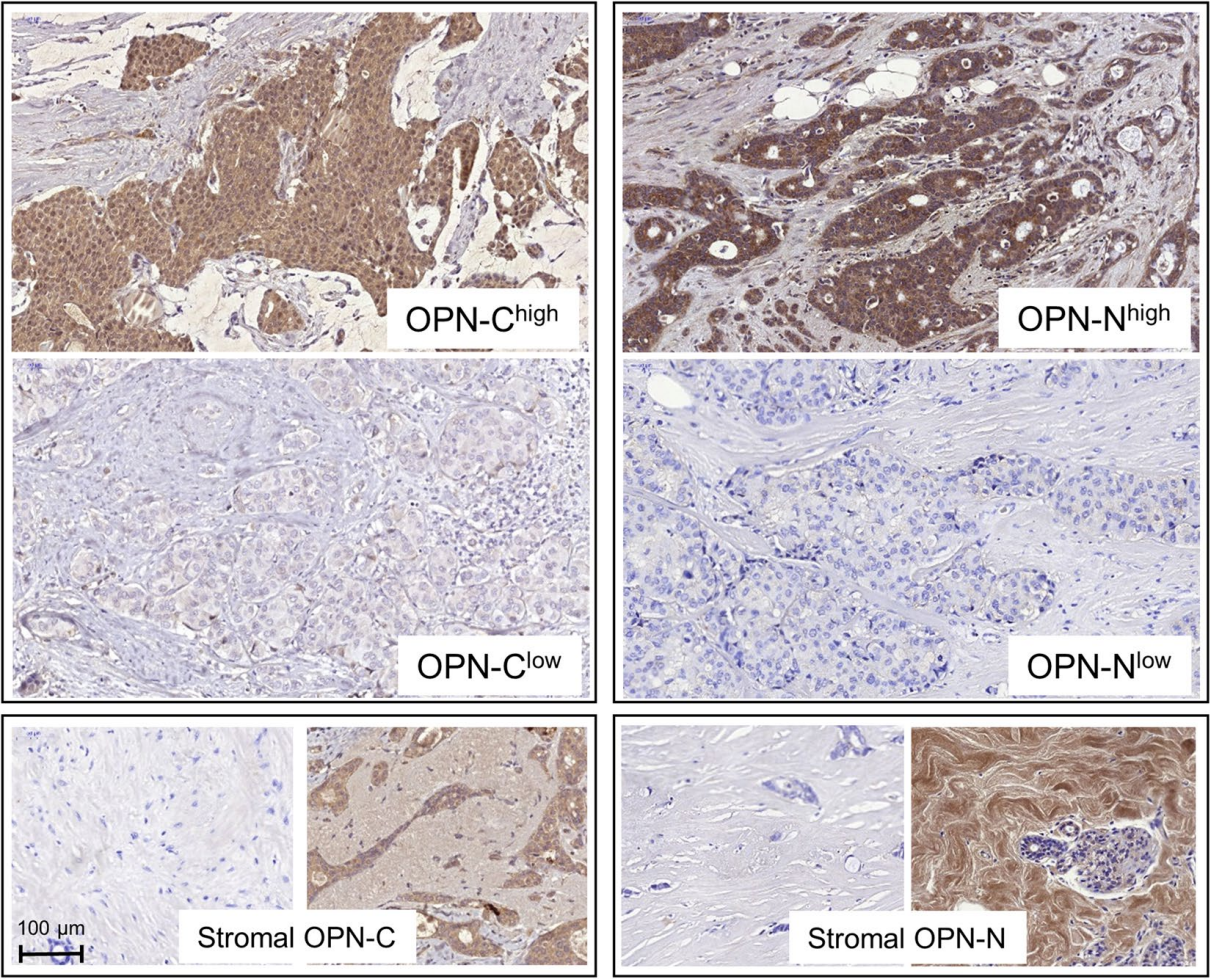
Micrographs of IHC staining using antibodies against OPN C and N-terminals. All micrographs are snapshots taken with CaseViewer (3DHistech) in x20 magnification. The bottom left scale bar applies to all micrographs.

**Figure 4:**
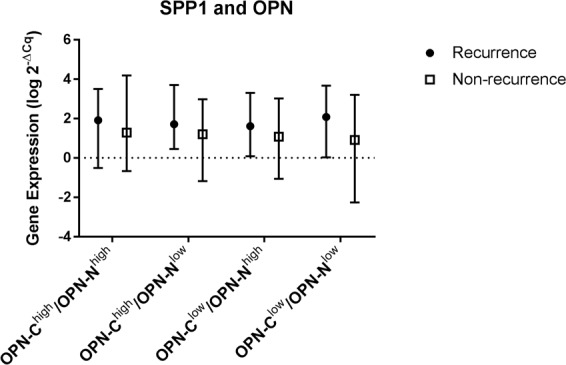
Graph showing the distribution of gene expression among tumours from breast cancer patients with and without distant metastasis (recurrence), grouped according to pattern of OPN-C/OPN-N protein expression. The symbol represents the median values with whiskers showing minimum and maximum gene expression values.

OPN-staining was assessed for 116 patients, described in Table 1. The staining was located to the cytoplasm of the tumour cells and to the stroma (Fig. 3). Staining controls are shown in Supplementary Figure 3. H-score for tumour cell expression of OPN-C ranged from 30 to 247 (median 106), and H-score for tumour cell expression of OPN-N ranged from 0 to 265 (median 13). The distribution of patients in the H-score 20^th^ percentile groups is described in Table 2. The majority of the tumours showed low expression of stromal OPN-C, categorized as 0 (n = 79, 68.1%) and 1 (n = 37, 31.9%). For stromal OPN-N, patients were distributed in all four staining categories; 0 (n = 85, 73.3%), 1 (n = 25, 21.6%), 2 (n = 5, 4.3%) and 3 (n = 1, 0.9%). Shown by Kruskal Wallis test, tissues with stromal staining of OPN-N ( ≥ 1) had more often higher tumour cell expression of OPN-N (OPN-C^low^/OPN-N^high^ and OPN-C^high^/OPN-N^high^, *p* < 0.0001) while tissues with stromal OPN-C were equally distributed between the different categories of tumour cell expression. None of the staining categories were associated with recurrence (Chi2-test) or to risk of recurrence (conditional regression analysis and 20^th^ percentile H-scores, Table 3, Table 4 and Fig. 4). The results from the additional log-rank survival tests show no association of OPN-C or OPN-N expression to shorter recurrence-free survival, presented in Kaplan-Meier curves in Supplementary Figure 2.

**Table 2:** The distribution of patients (n = 116) by tumour cell OPN-C and OPN-N protein expression by H-scores grouped by 20th percentiles. The lowest 20th percentile H-scores is represented by number 1 and the highest 20th percentile H-scores by 5.

	Number (%) of patients with OPN-C						
		1	2	3	4	5	Total
Number (%) of patients with OPN-N	1	7 (6.0)	4 (3.4)	7 (6.0)	3 (2.6)	2 (1.7)	23
	2	4 (3.4)	6 (5.2)	7 (6.0)	7 (6.0)	3 (2.6)	27
	3	6 (5.2)	3 (2.6)	2 (1.7)	4 (3.4)	5 (4.3)	20
	4	4 (3.4)	4 (3.4)	4 (3.4)	5 (4.3)	6 (5.2)	23
	5	2 (1.7)	6 (5.2)	3 (2.6)	5 (4.3)	7 (6.0)	23
Total		23	23	23	24	23	116

**Table 3:** The tumour cell OPN staining categorized by the median* H-score into high- and low.

	Number (%) of patients			
	OPN-C^high^		OPN-C^low^	
	Recurrence	Non-recurrence	Recurrence	Non-recurrence
OPN-N^high^	6 (5.2)	28 (24.1)	6 (5.2)	10 (8.6)
OPN-N^low^	5 (4.3)	19 (16.4)	10 (8.6)	21 (18.1)

*Median H-score for OPN-C = 106, Median H-score för OPN-N = 13.

**Table 4:** Risk of recurrence by C- and N-terminal OPN in tumour cells and stroma. The table shows 95% confidence intervals (CI) for the odds ratio (OR).

	Score	Odds ratio (OR)	95% CI	P-value
OPN-C	1	1		
	2	1.26	0.33–4.79	0.73
	3	0.34	0.06–1.83	0.21
	4	0.23	0.04–1.31	0.10
	5	0.48	0.08–2.71	0.41
OPN-N	1	1		
	2	1.76	0.39–8.00	0.46
	3	< 0.0001	0 -∞	0.99
	4	1.54	0.41–5.78	0.53
	5	1.01	0.22–4.64	0.99
OPN-C, Stroma	0	1		
	1	1.01	0.38–2.67	0.99
OPN-N, Stroma	0	1		
	1	0.93	0.32–2.71	0.90
	2	0.70	0.06–8.10	0.77
	3	< 0.0001	0 -∞	0.99

### Correlation between gene- and protein expression

**Figure 5:**
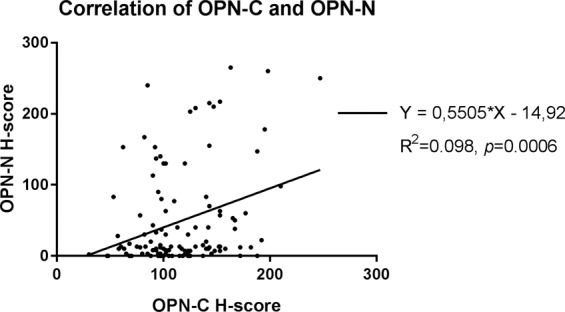
Correlation between staining results (H-score) of OPN-C and OPN-N expression in tumours. The scatter plot illustrates values for each individual tumour (n = 116).

There were no correlations between tumour cell OPN-C and SPP1 (Spearmans r = 0.003, *p* = 0.98) or tumour cell OPN-N and SPP1 (Spearmans r = −0.047, *p* = 0.65) and regression analysis show no relationships of the variables of gene- and protein expression (R^2^ > 0.01). The OPN-C and OPN-N expressions (H-score) were however correlated (Spearmans r = 0.24, *p* = 0.009) but linear regression analysis indicated a low magnitude association (R^2^ = 0.098, *p* = 0.0006, Fig. 5). The gene expression of SPP1 did not differ between cases with OPN-C^high^/OPN-N^high^, OPN-C^high^/OPN-N^low^, OPN-C^low^/OPN-N^high^ or OPN-C^low^/OPN-N^low^ (*p* = 0.74, Fig. 4).

## Discussion

In this case-control study, we demonstrated that high SPP1 gene expression was significantly associated with increased risk of distant recurrence among tamoxifen- treated women with ER+ breast cancer. A similar study associating SPP1 with worse outcome showed that breast tumours with high SPP1 mRNA expression were more often ER/PR-negative^19^. If there is an association between SPP1 and lower ER signalling, patients with an ER+ tumour that expresses higher levels of SPP1 could theoretically be less responsive to anti-oestrogen treatment. However, the SPP1 mRNA had no value in terms of predicting tamoxifen benefit^19^. We measured SPP1 gene expression with qPCR using primers and probes that detect all five known mRNA transcripts of SPP1. We used a matched case-control design and report that the risk of recurrence for tamoxifen- treated breast cancer patients increases with higher SPP1 expression. The results from mRNA analyses strongly suggest that functional studies are needed in order to find the underlying biological background for SPP1 behaviour in ER+ breast cancer. We also aimed to investigate if SPP1 protein expression, OPN, may have predictive value for recurrence after endocrine treatment. Oestrogen functions as a growth factor in these tumours, stimulating cell proliferation and growth by binding to ER. Tamoxifen competes with oestrogen for ER-binding, explaining why high levels of oestrogen may reduce the effect of treatment^1^. OPN (cleaved by MMPs) has been shown to induce the expression of aromatase (*CYP19A1*), an enzyme involved in oestrogen biosynthesis^20^ and could thereby contribute to tamoxifen resistance by increasing oestrogen production. Moreover, OPN upregulate multiple genes including INSIG1, CTFG and CYR61. These genes have been reported to provide breast cancer cells with self-sufficiency of growth signals, making them less dependent of oestrogen^21^. High expression of OPN could hypothetically induce growth of cancer cells regardless of tamoxifen treatment. We could however not detect evidence that OPN expression predicts recurrence, estimated by IHC staining of both C- and N terminals in tumour cells and in tumour stroma. Even though OPN has proven ability to predict recurrence, its clinical implications would be limited since OPN exists in diverse isoforms and is subject to cleavage and posttranslational modifications [1], making the protein expression hard to interpret. The anti OPN-C antibody used in present study detects all isoforms of OPN, while the anti OPN-N antibody detects only OPN-a and OPN-b. Some studies report conflicting evidence, but as there is no referral to specific isoform, the results from these studies are difficult to evaluate^19,22,23^. Interestingly, recent evidence suggests that exon 4 (lacking from OPN-c) is associated with favourable response to tamoxifen^2^ but studies regarding the prognostic value of OPN have identified splice variant OPN-c to be more reliably associated with prognosis than full-length OPN^15^. A recent study by Walaszek *et al*. confirmed that high levels of OPN-c is related to unfavourable prognosis^24^.

In order to evaluate predictive or prognostic values of OPN, the different splice variants require separate detection. It is questionable whether immunohistochemistry is specific enough to determine the certain isoforms as the antibody binding may be affected by several posttranslational modifications. Methodological effects could also explain why we were not able to demonstrate any correlations between gene- and protein expressions. Gene expressions by qPCR reflect the tissues’ total amount of mRNA (all transcripts), while the evaluation of IHC staining was restricted to tumour cells and stroma by looking at two parts of the protein. Another potential confounding factor relevant to detection of OPN protein relates to the presence of both intracellular and secreted forms. The intracellular form of OPN is retained in the cytoplasm due to absence of the signal sequence necessary for translocation over the ER membrane and subsequent secretion. The relative abundance and cellular half-life of the secreted and intracellular forms appears to be cell-type specific^25^, which could influence the steady-state levels of OPN protein in comparison with SPP1 gene expression. Also, little is known regarding the post-translational modifications including phosphorylation, glycosylation and proteolytic processing occurring in the intracellular form of OPN, potentially confounding comparison between the different isoforms.

Given the different functions, isoforms, posttranslational modifications and cellular origins of OPN, that could potentially influence antibody-based detection methods, we conclude that SPP1 mRNA is currently a more stable and reliable indicator of expression. More studies and additional methods of detection are needed to elucidate the predictive value of SPP1, also in comparison to established prognostic markers e.g. Ki-67, and any applicability in clinical practice.

## Conclusion

Higher SPP1 gene expression in primary tumours was found to be associated with risk of recurrence in ER+ breast cancer among patients with endocrine treatment, while OPN protein expression does not appear to be predictive of recurrence.

## Supplementary information

Supplementary Information.

## Data Availability

All data generated and analysed during the current study is available from the corresponding author on reasonable request.
