# Supplementary material for: Evaluation of SPP1/osteopontin expression as predictor of recurrence in tamoxifen treated breast cancer
Source: Sci Rep. 2020 Jan 29;10:1451. doi: 10.1038/s41598-020-58323-w (PMC6989629; doi:10.1038/s41598-020-58323-w)
Supplement: Supplementary file 1 — Supplementary Information. [file 41598_2020_58323_MOESM1_ESM.docx]

Supplementary Information

**Evaluation of SPP1/osteopontin expression as predictor of recurrence in tamoxifen treated breast cancer**

*Göthlin Eremo Anna^1,2^, Lagergren Kajsa^2^, Othman Lana^2^, Montgomery Scott,^3 4 5^ Andersson Göran^6^, Tina Elisabet^1^,

1. Department of Clinical Research Laboratory, Faculty of Medicine and Health, Örebro University, Örebro, Sweden
2. School of Medical Sciences, Faculty of Medicine and Health, Örebro university, Örebro, Sweden
3. Clinical Epidemiology and Biostatistics, School of Medical Sciences, Örebro University, Örebro, Sweden
4. Clinical Epidemiology Division, Karolinska Institutet, SE-171 76 Stockholm, Sweden
5. Department of Epidemiology and Public Health, University College London, 1-19 Torrington Place, London, WC1E 7HB, United Kingdom
6. Division of Pathology, Department of Laboratory Medicine, Karolinska Institutet and Karolinska University Hospital Huddinge, S-141 86 Huddinge Sweden

*Corresponding author: Anna Göthlin Eremo,

E-mail:anna.gothlin-eremo@regionorebrolan.se, Telephone +46 (0)19 6026652

Address: Clinical Research Laboratory, Campus USÖ, 3th floor, Örebro University Hospital. SE-70185 Örebro, Sweden

Figure 1:


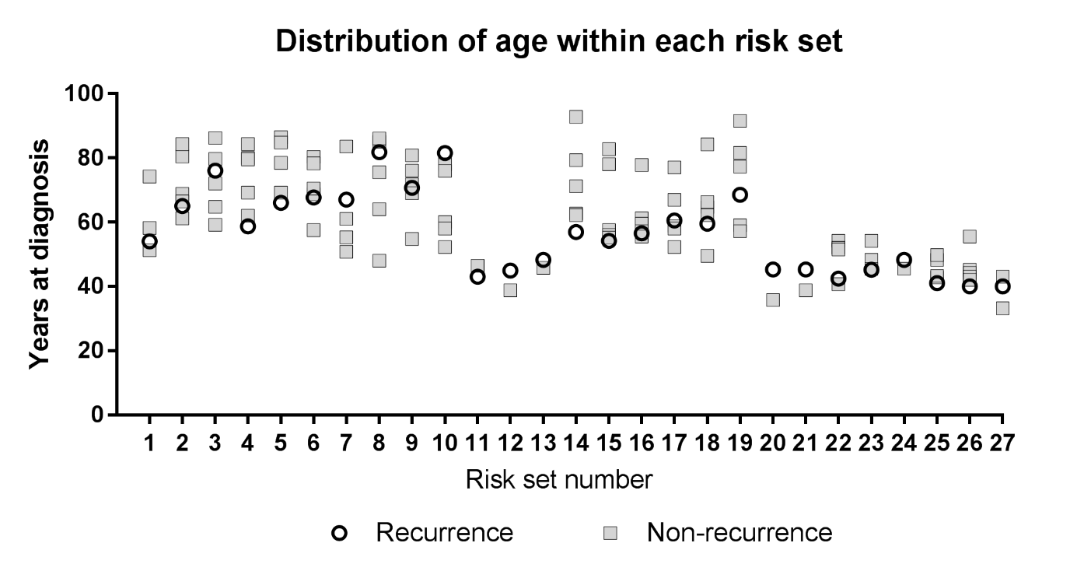


Figure 1. The recurrence patients are younger than the non-recurrence patients. However, when looking at the age distribution in each risk set, the recurrence patient is youngest in only 7 out of 27 sets.

Figure 2:


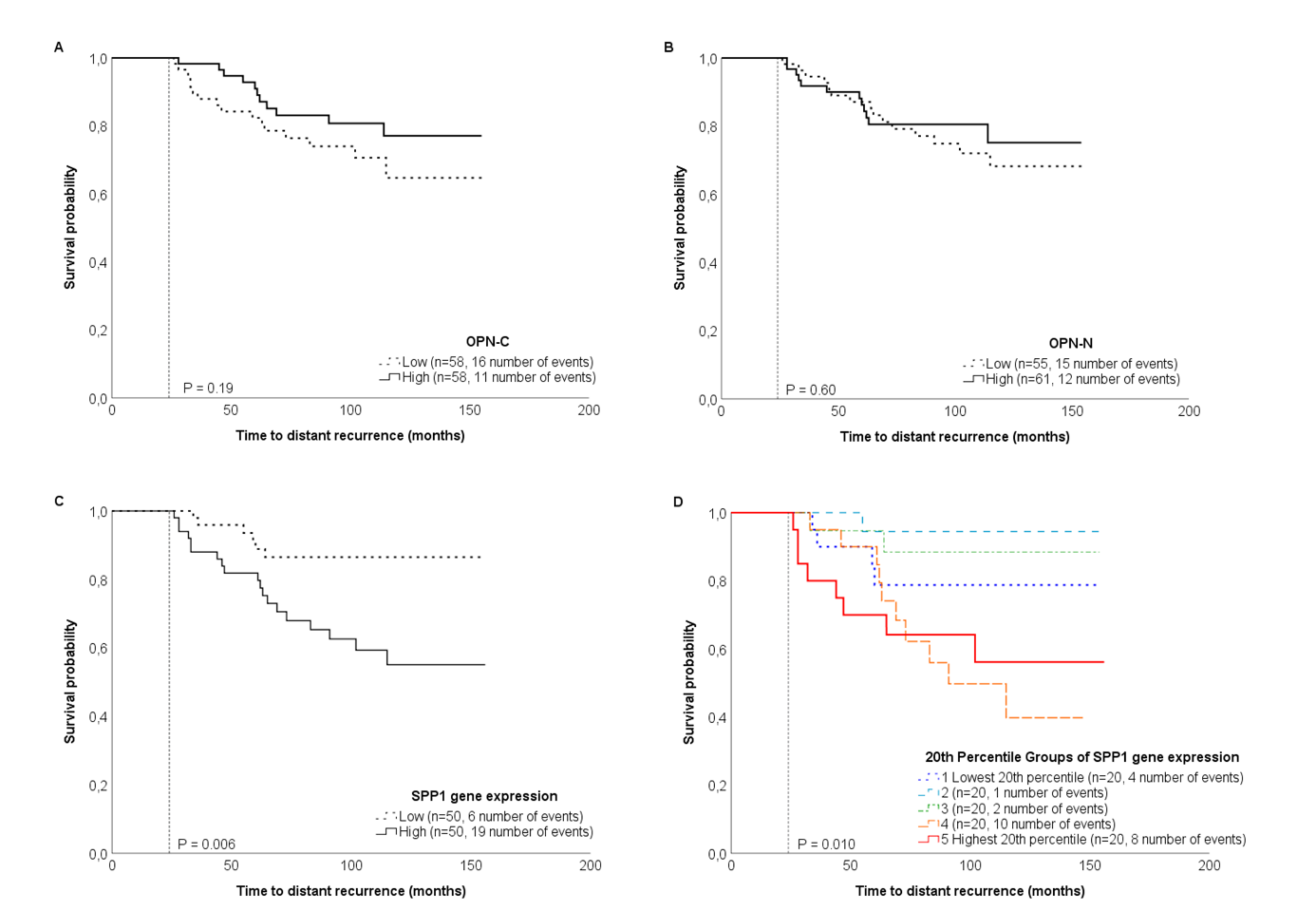


Figure 2. Kaplan-Meier survival curves for breast cancer patients with different tumour levels of OPN protein- and SPP1 gene expression in the matched case-control study. All patients were recurrence-free at least 24 months from primary diagnosis; the vertical dotted reference line refers to this time point. In the log-rank test, low and high define as below or above the median H-score OPN protein expression (A and B) or median Z-Score SPP1 gene expression (C) respectively. There were no differences in survival between patients with tumours categorised as having high or low OPN-C (A) or OPN-N (B) protein expression. However, patients with high SPP1 gene expression have a significantly shorter time to recurrence than those with low gene expression (C). Also, when the Z-scores were ordered numerically and divided into five equal sized groups based on 20^th^ percentiles, the two groups with the highest gene expression had the shortest time to recurrence (D).

Figure 3:


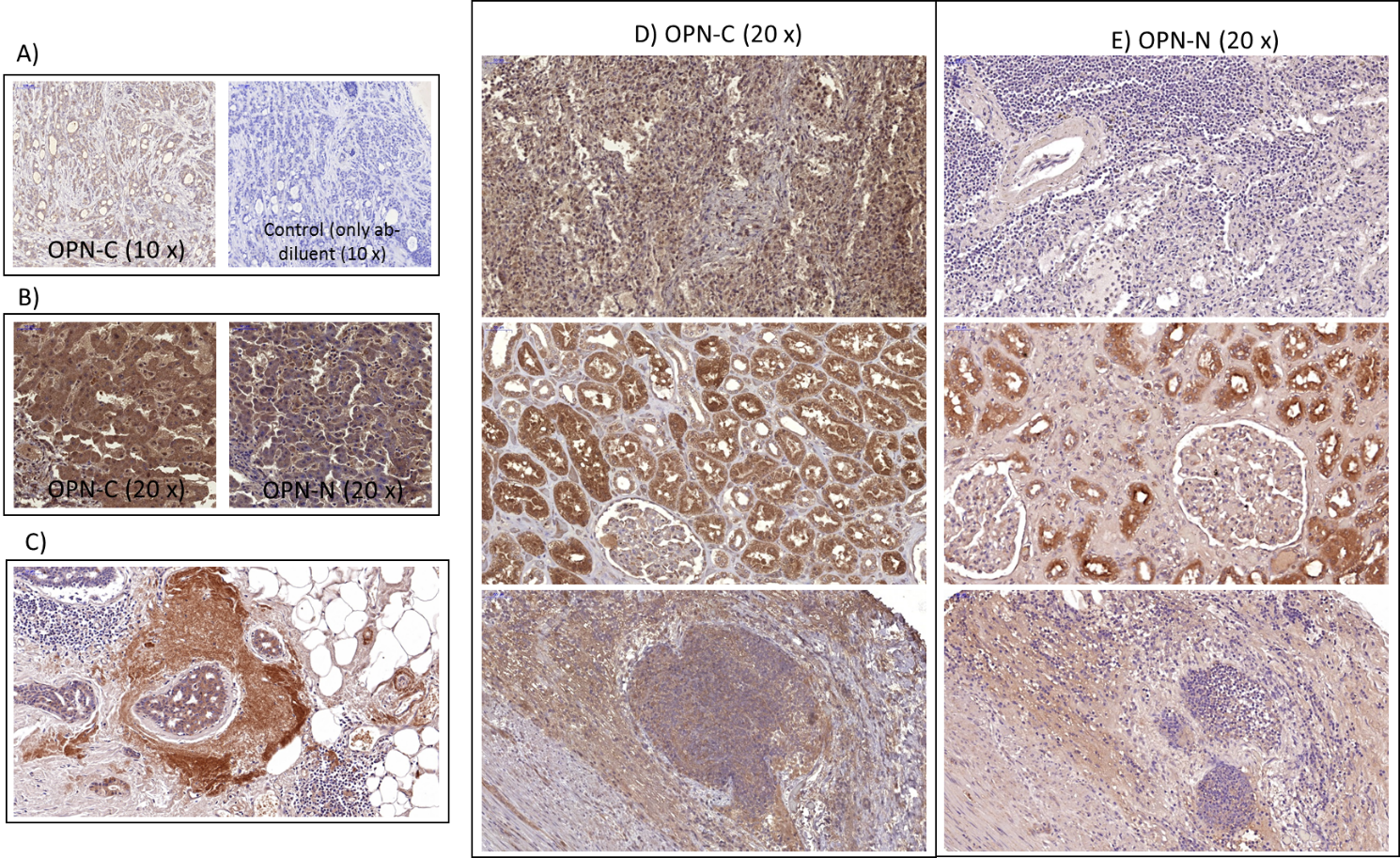


Figure 3: Images of OPN-stainings A) Breast cancer tissue from one patient incubated with OPN-C antibody (left) in comparison to staining control (diluent-only, right). B) Liver tissue stained with OPN-C (left) and OPN-N (right) antibody showing different expression pattern. C) Breast stroma with a strong positive OPN-N immunoreaction (brown), surrounding cancer cells. The lesser-stained stromal compartment surrounding adipose cells suggests less OPN-N expression. D) Selected tissues showing OPN-C (left column) and OPN-N (right column)-staining. From above; spleen, kidney and lymphoid aggregates in appendix. As described by Brown, L. F. *et al*. (Expression and distribution of osteopontin in human tissues: widespread association with luminal epithelial surfaces. Mol Biol Cell 3, 1169-1180, doi:10.1091/mbc.3.10.1169), OPN expression is located in epithelial luminal surfaces. OPN-C and OPN-N are expressed with equal staining intensity in kidney tubules (Figure 3D, second images from the top).

Table 1:

| Table 1. The diagnostic ability of OPN gene expression (z-scores) was evaluated using Reciever Operating Characteristic (ROC) curve analysis, with Area Under the Curve (AUC), in comparison to other known diagnostic and prognostic markers (Elston grade, tumour size, lymph nodes and HER2-status. The non-significant and low AUC values for the other markers are most likely a result from using matched patients. | | | | | |
| --- | --- | --- | --- | --- | --- |
| Test Result Variable(s) | Area | Std. Error | Asymptotic Sig.  (*p*-value) | Asymptotic 95% Confidence Interval | |
|  |  |  |  | Lower Bound | Upper Bound |
| Elston grade | 0.526 | 0.069 | 0.705 | 0.391 | 0.661 |
| SPP1 (z-value) | 0.702 | 0.063 | 0.003 | 0.578 | 0.825 |
| Tumour size (mm) | 0.523 | 0.072 | 0.736 | 0.381 | 0.665 |
| Lymph nodes (Pos/Neg) | 0.515 | 0.069 | 0.827 | 0.380 | 0.650 |
| HER2-status | 0.494 | 0.069 | 0.935 | 0.359 | 0.629 |
